# Supplementary material for: Structural basis of adhesion GPCR GPR110 activation by stalk peptide and G-proteins coupling
Source: Nat Commun. 2022 Sep 20;13:5513. doi: 10.1038/s41467-022-33173-4 (PMC9489763; doi:10.1038/s41467-022-33173-4)
Supplement: Supplementary file 1 — Supplementary Information [file 41467_2022_33173_MOESM1_ESM.pdf]

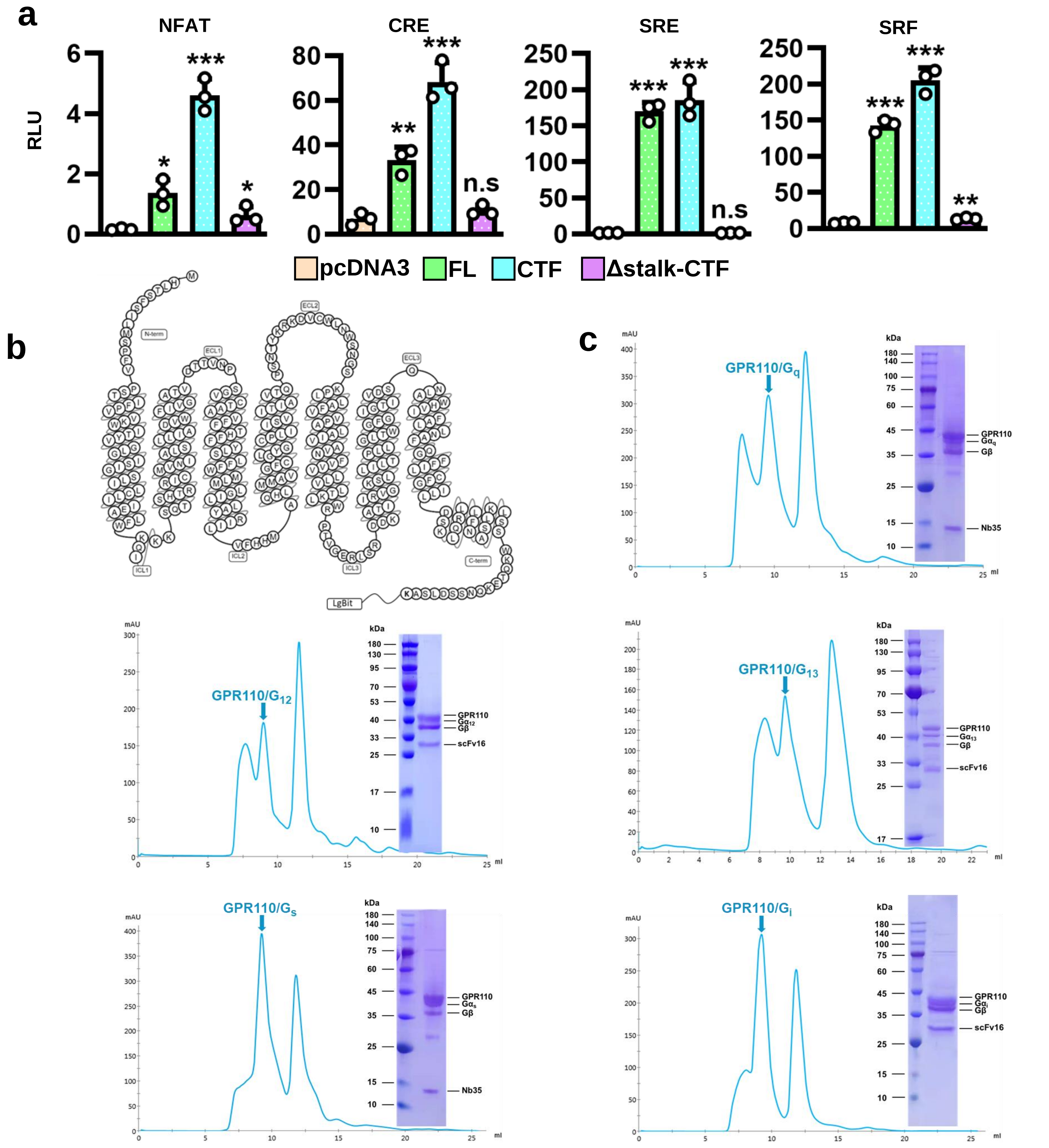

Supplementary Fig. 1. GPR110 is able to couple with all 4 major G-protein signaling pathways. a, Reporter assays of GPR110 constructs. FL, full-length. CTF, C-terminus fragment. RLU, relative luciferase unit. Data are presented as mean values ± SD; n =3 independent samples. n.s. no significant; *, $P < 0.05$; **, $P < 0.01$; ***, $P < 0.001$. Data between WT and mutants were analyed by two-sided test (from left to right for NFAT group, $P = 0.0444$, $P < 0.001$ and $P = 0.0457$; from left to right for CRE group, $P = 0.0021$, $P < 0.001$ and $P = 0.1498$; from left to right for SRE group, $P < 0.001$, $P < 0.001$ and $P = 0.1755$; from left to right for SRF group, $P < 0.001$, $P < 0.001$ and $P = 0.0078$).Source data are provided as a Source Data file. **b**, Snake-shape diagram of the GPR110 construct used in complex assembling, the diagram was adopted from GPCRdb. **c**, Size exclusion column profile of GPR110/G-protein complex. Experiments were repeated independently 3 times with similar results. Source data are provided as a Source Data file.

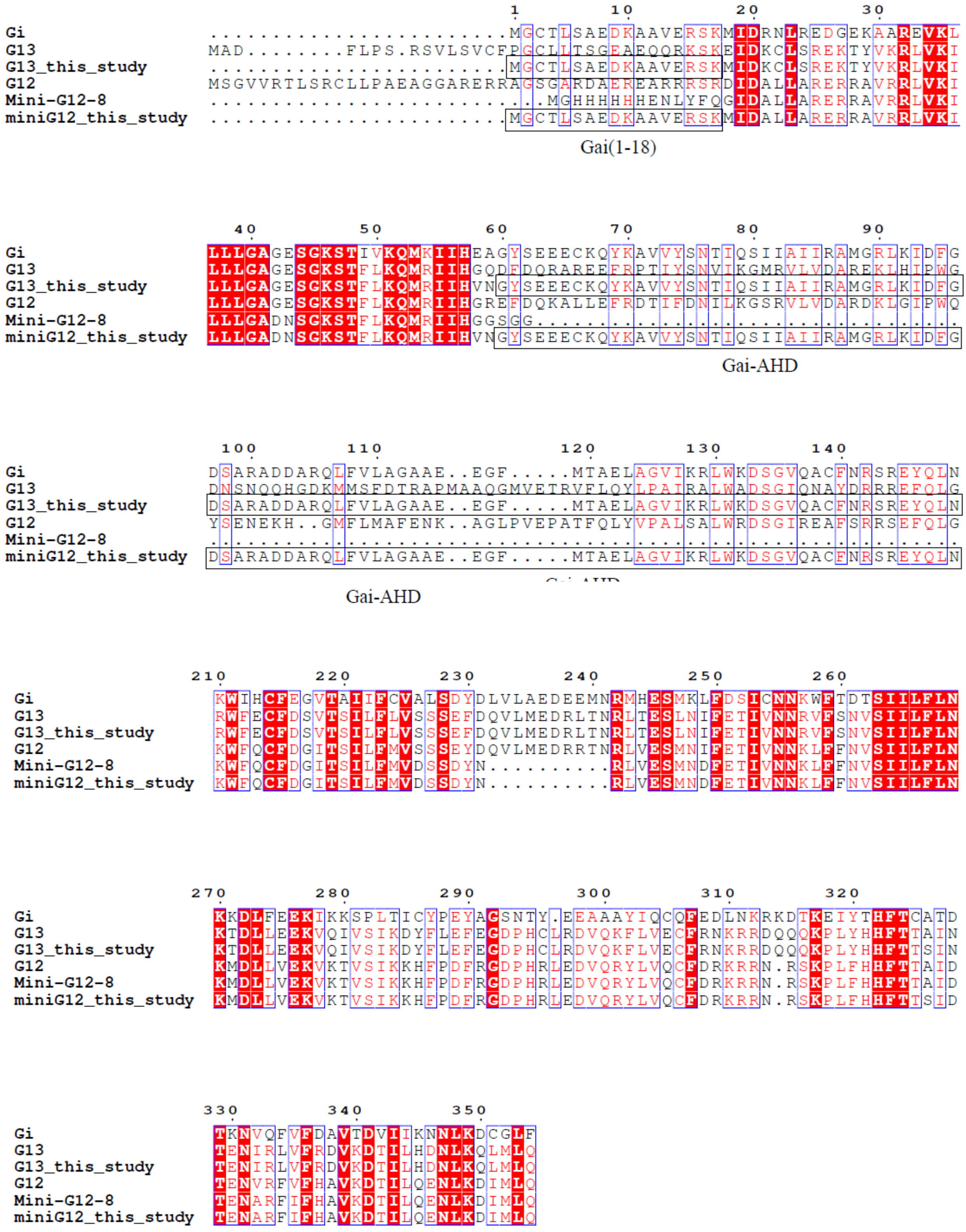

Supplementary Fig. 2. An alignment of Gα_{12} and Gα_{13} construct used in this study.

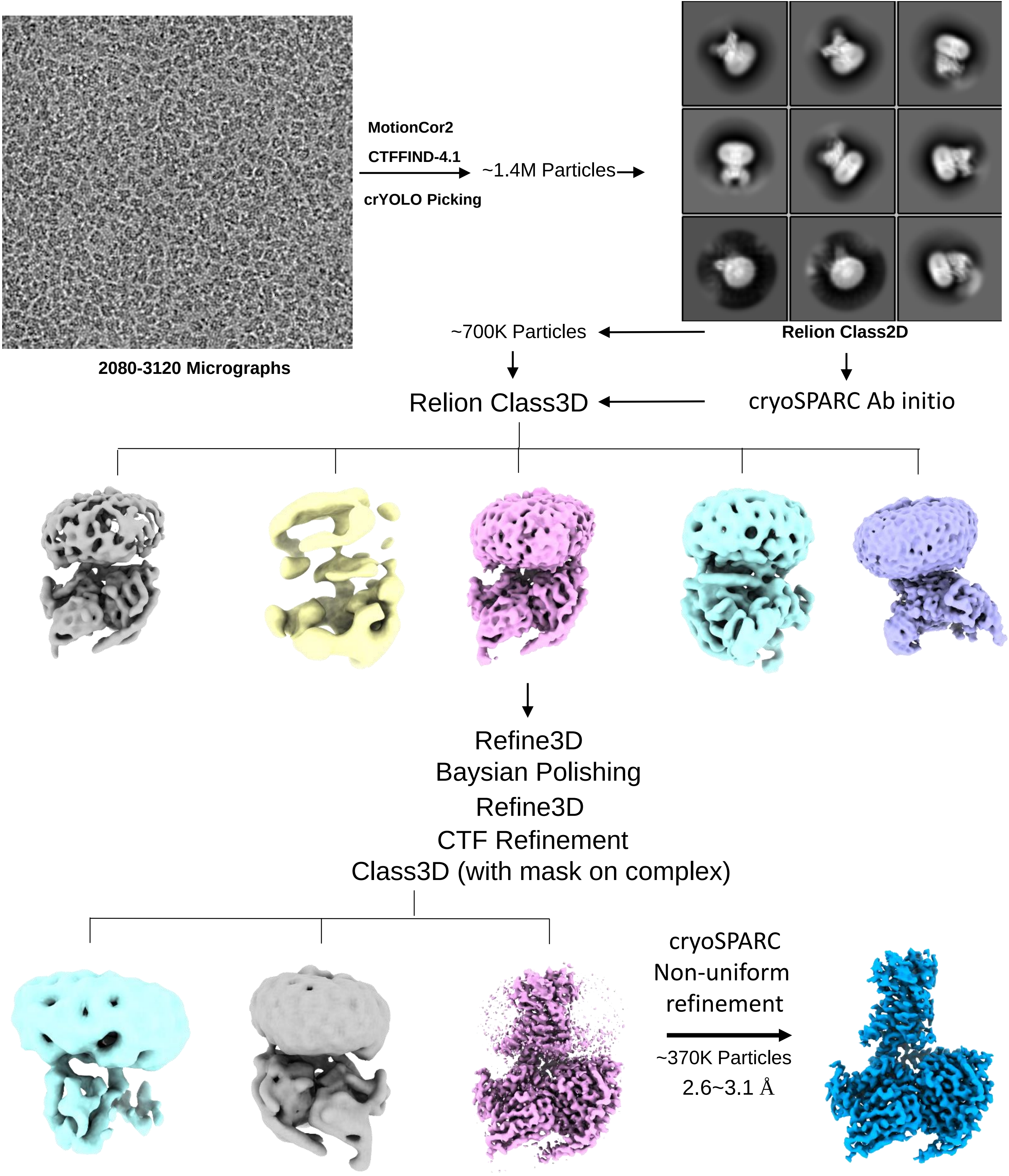

Supplementary Fig. 3. Flow-chart of cryo-EM data process of GPR110/G-protein complexes.

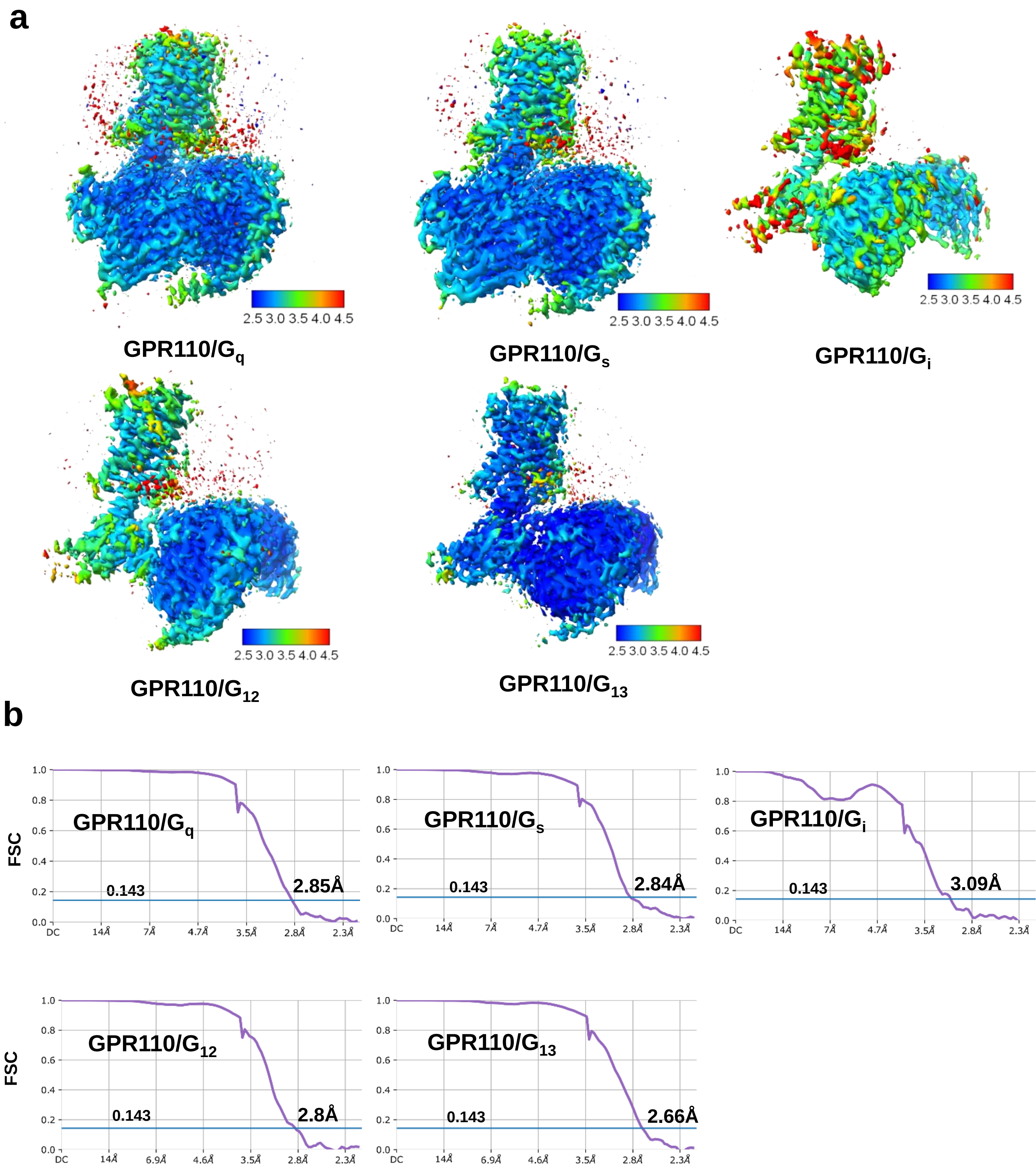

Supplementary Fig. 4. Resolution of GPR110/G-protein complexes. a, Local resolution analysis of GPR110/G-protein complexes. **b**, FSC curve of the GPR110/G-protein complexes, the resolution was assessed by the Gold Standard of FSC=0.143.

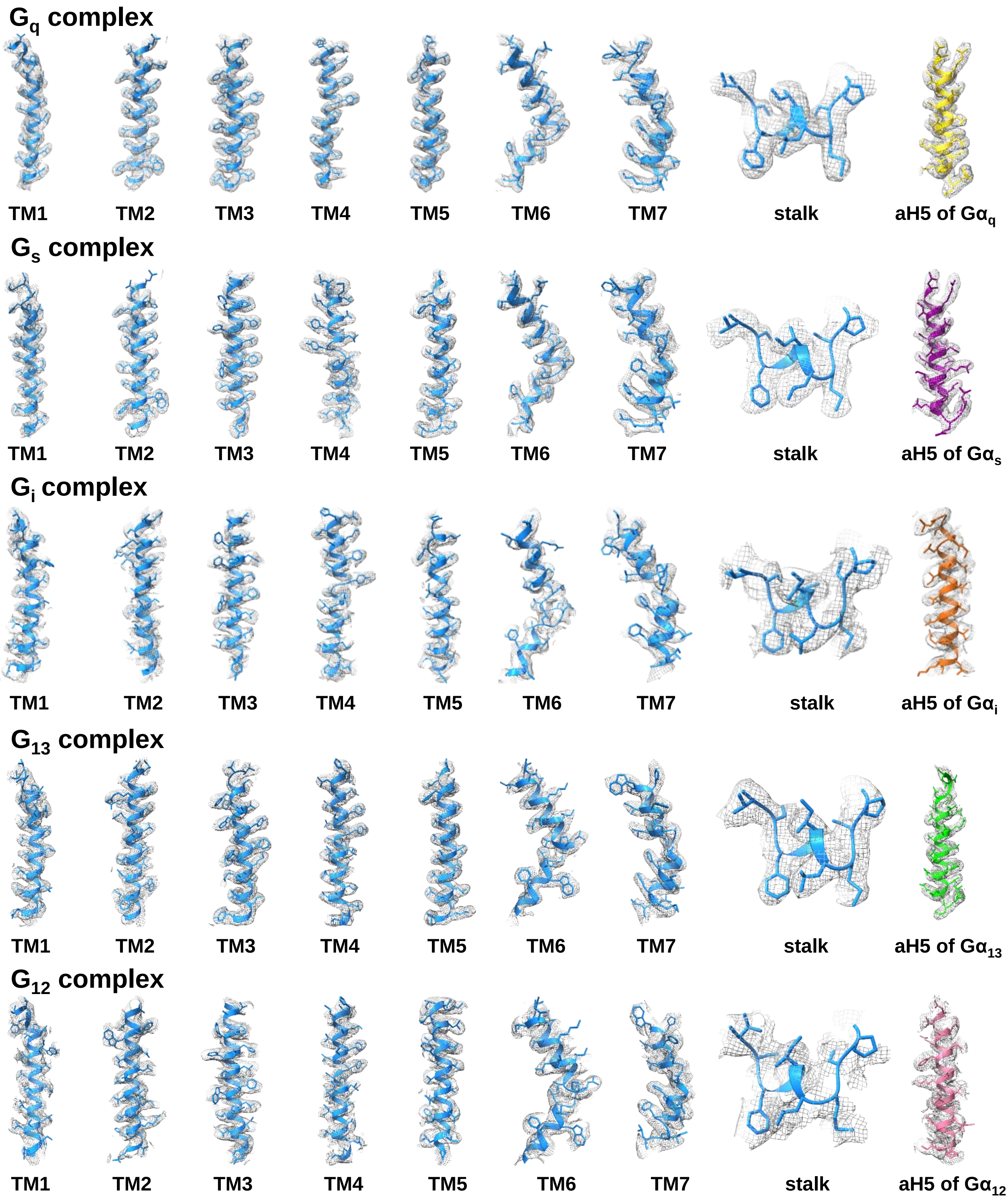

Supplementary Fig. 5. Cryo-EM density map of representative regions of GPR110/G-protein complex. Contour level of 0.08.

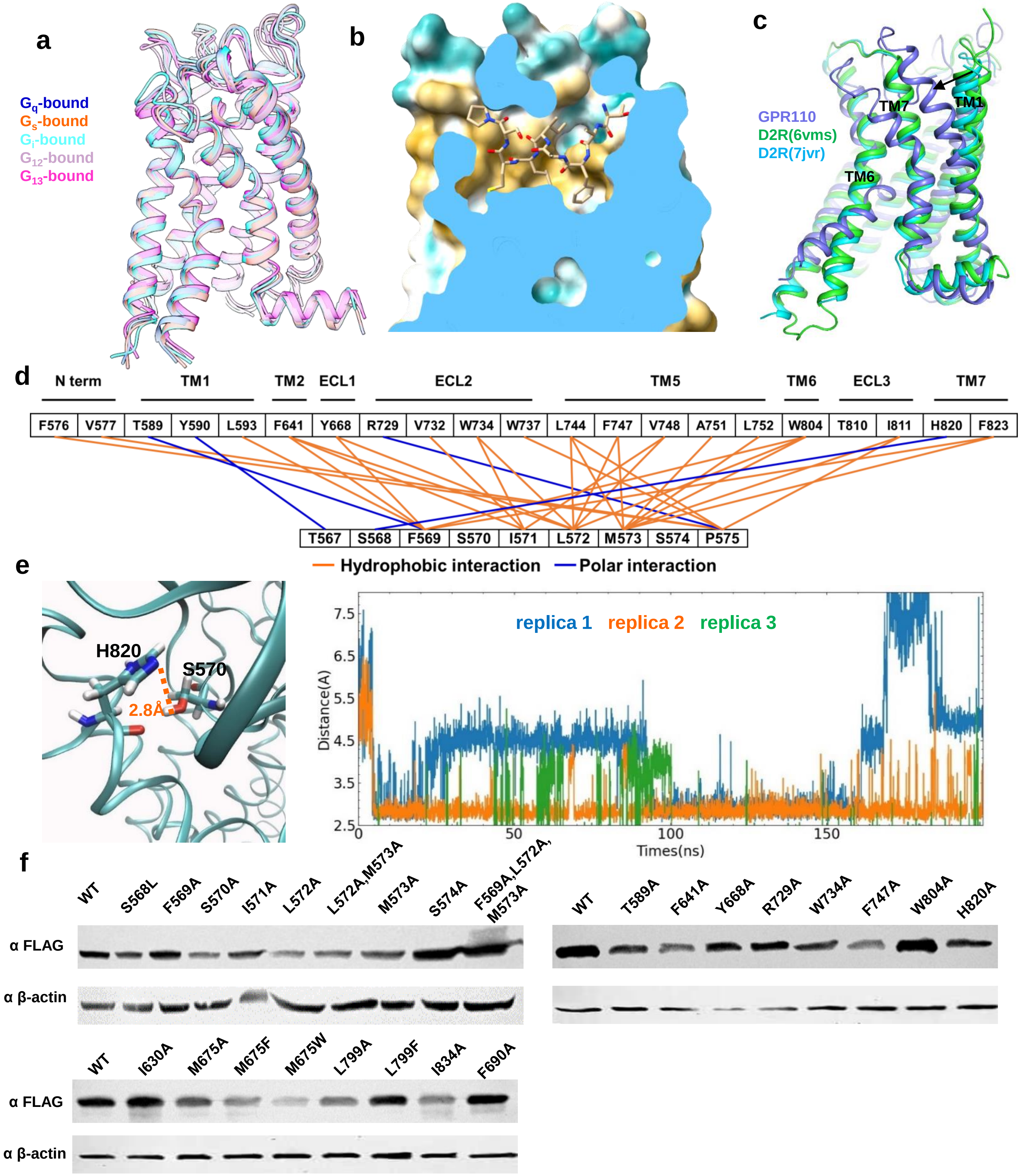

Supplementary Fig. 6. Addtional information of the ligand binding pocket of GPR110. **a**, A comparison of G_q-bound, G_s-bound, G_i-bound, G_{12}-bound and G_{13}-bound GPR110. **b**, Hydrophobicity analysis of the ligand binding pocket of GPR110. **c**, A comparison of GPR110 with D2R. **d**, The interaction map of the stalk peptide ligand/receptor interaction of GPR110. **e**, MD simulation analysis of S570^{stalk}/H820$^{7.39}$ interaction. Left panel, a snapshot of S570^{stalk}/H820$^{7.39}$ interaction in a MD simulation of active GPR110; right panel, trajectory analysis of S570^{stalk}/H820$^{7.39}$ interaction. **f**, Western-blot of GPR110 mutants in this study. The C-terminus of GPR110 was fused to a FLAG tag. Source data are provided as a Source Data file. Experiments were repeated independently 3 times with similar results.

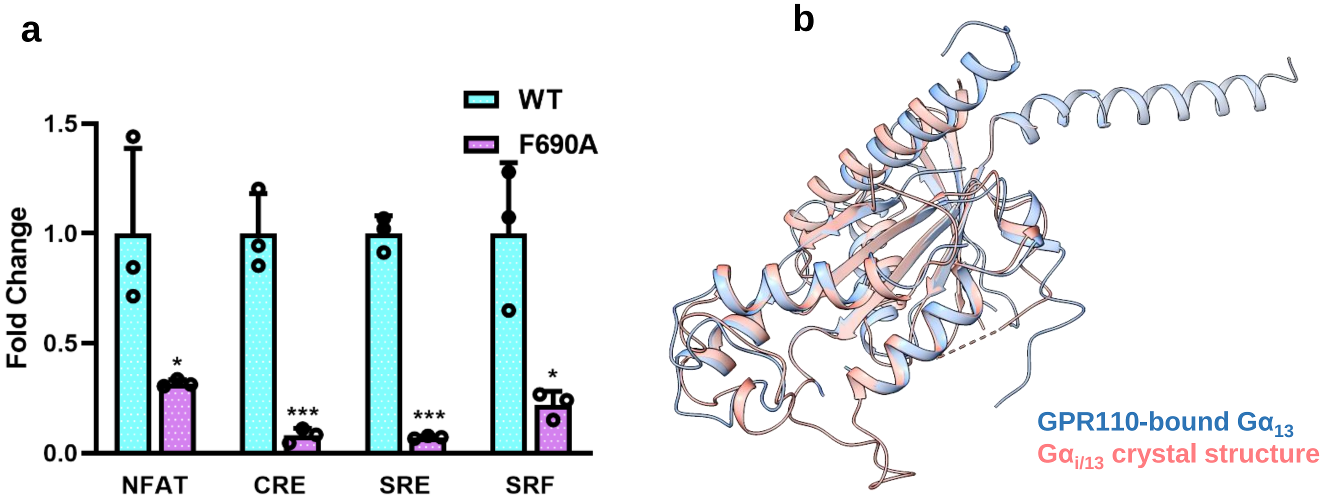

Supplementary Fig. 7. Additional information of G_{12} G_{13} engagements of GPR110 activation. a, F690^{ICL2}A decrease receptor activation in reporter assay. Data are presented as mean values ± SD; n =3 independent samples. *, $P < 0.05$; ***, $P < 0.001$. Data between WT and mutants were analyzed by two-sided test (for NFAT group, $P = 0.0379$; for CRE group, $P < 0.001$; for SRE group, $P < 0.001$; for SRF group, $P = 0.0144$). Source data are provided as a Source Data file. **b**, A comparison of GPR110-bound $G\alpha_{13}$ with the crystal structure of $G\alpha_{i/13}$ (PDB 1zcb).

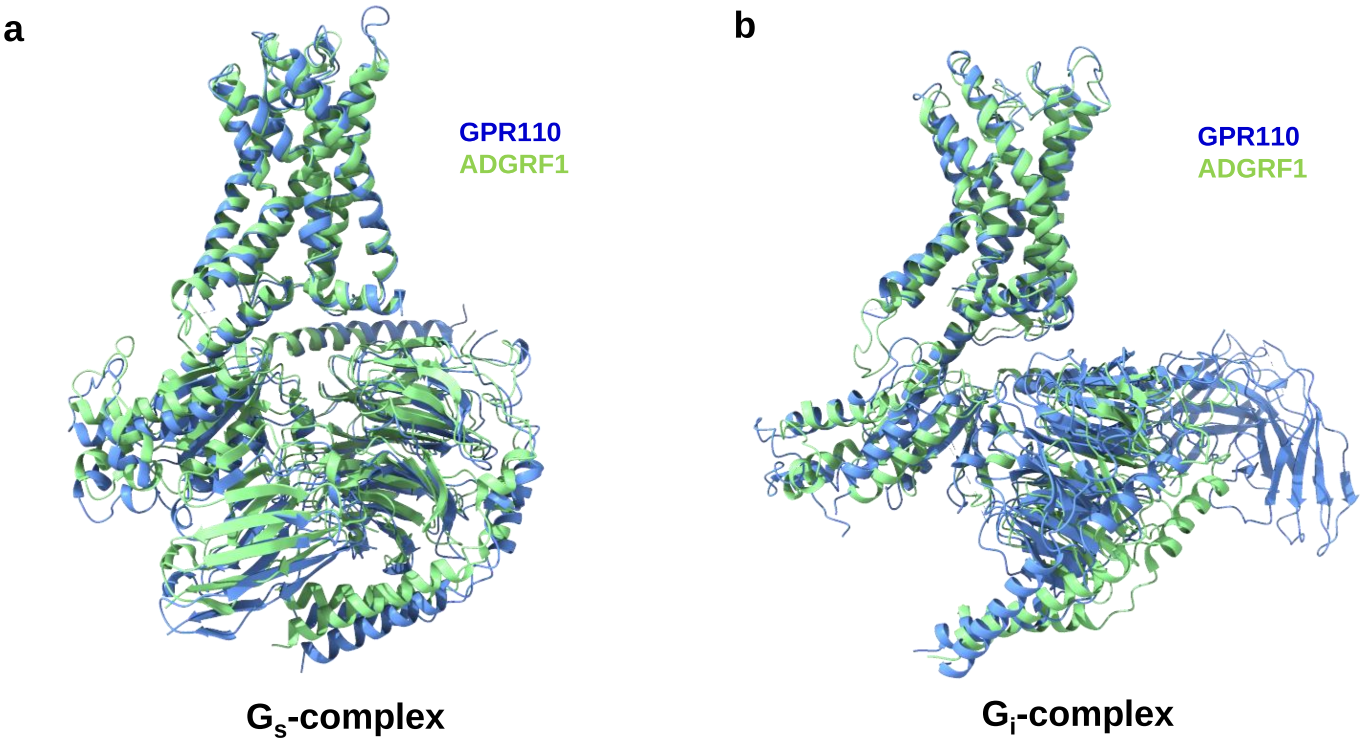

Supplementary Fig. 8. Comparisons of GPR110 with published ADGRF1 G-protein complex structures. a, A comparison of G_s engagements between GPR110 and ADGRF1 (7wu3). **b**, A comparison of G_i engagements between GPR110 and ADGRF1 (7wu5).

Supplementary Table 1. Cryo-EM data collection and refinement statistics

	GPR110/G_q EMD-32881 7WXU	GPR110/G_s EMD-32882 7WXW	GPR110/G_i EMD-32972 7X2V	GPR110/G_{12} EMD-32905 7WZ7	GPR110/G_{13} EMD-32883 7WY0
**Data collection and processing**					
Magnification	130,000	130,000	130,000	130,000	130,000
Voltage (kV)	300	300	300	300	300
Electron exposure ($e^-/Å^2$)	60	60	60	60	60
Defocus range (μm)	1.2-2.2	1.2-2.2	1.2-2.2	1.2-2.2	1.2-2.2
Pixel size (Å)	0.55	0.55	0.55	0.54	0.54
Symmetry imposed	C1	C1	C1	C1	C1
Initial particle image (no.)	1.7M	2.4M	2.1M	4.0M	3.8M
Final particle image (no.)	330k	540k	260k	540k	610k
Map resolution (Å)	2.85	2.84	3.09	2.8	2.66
FSC threshold	0.143	0.143	0.143	0.143	0.143
**Refinement**					
Initial model used (PDB code)	AF-Q5T601-F1, 7f4d	AF-Q5T601-F1, 7f4d	AF-Q5T601-F1, 6vms	AF-Q5T601-F1, 6vms	AF-Q80TS3-F1, 6vms
Model Resolution (Å)	3.3	3.3	3.3	3.3	3.3
FSC threshold	0.143	0.143	0.143	0.143	0.143
Map sharpening *B* factor ($Å^2$)	-118.6	-122.4	-109.1	-125.7	-107.9
Model composition					
Non-hydrogen atoms	8116	8121	8761	8676	8724
Protein residues	1034	1032	1123	1108	1112
Ligands	0	0	0	0	0
B factor ($Å^2$)					
Protein	43.68	42.53	30	30	28.3
Ligand					
R.m.s. deviations					
Bond length (Å)	0.009	0.006	0.008	0.01	0.008
Bond angles (°)	1.148	1.088	1.174	1.204	1.146
Validation					
MolProbity score	1.2	1.15	1.68	1.7	1.79
Clashscore	1.05	1.23	5.54	6.04	6.75
Poor rotamers (%)	0	0	0	0	0
Ramachandran plot					
Favored (%)	94.22	95.58	94.48	94.57	93.76
Allowed (%)	5.78	4.42	5.52	5.43	6.24
Disallowed	0	0	0	0	0
