# Supplementary material for: Structural basis of adhesion GPCR GPR110 activation by stalk peptide and G-proteins coupling
Source: Nat Commun. 2022 Sep 20;13:5513. doi: 10.1038/s41467-022-33173-4 (PMC9489763; doi:10.1038/s41467-022-33173-4)
Supplement: Supplementary file 3 — Reporting Summary [file 41467_2022_33173_MOESM3_ESM.pdf]

nature research

Corresponding author(s): Yuanzheng He

Last updated by author(s): Aug 21, 2022

Reporting Summary

Nature Research wishes to improve the reproducibility of the work that we publish. This form provides structure for consistency and transparency in reporting. For further information on Nature Research policies, see our Editorial Policies and the Editorial Policy Checklist.

Statistics

For all statistical analyses, confirm that the following items are present in the figure legend, table legend, main text, or Methods section.

n/a	Confirmed	
[ ]	[x]	The exact sample size (*n*) for each experimental group/condition, given as a discrete number and unit of measurement
[ ]	[x]	A statement on whether measurements were taken from distinct samples or whether the same sample was measured repeatedly
[ ]	[x]	The statistical test(s) used AND whether they are one- or two-sided<br>*Only common tests should be described solely by name; describe more complex techniques in the Methods section.*
[x]	[ ]	A description of all covariates tested
[x]	[ ]	A description of any assumptions or corrections, such as tests of normality and adjustment for multiple comparisons
[ ]	[x]	A full description of the statistical parameters including central tendency (e.g. means) or other basic estimates (e.g. regression coefficient) AND variation (e.g. standard deviation) or associated estimates of uncertainty (e.g. confidence intervals)
[ ]	[x]	For null hypothesis testing, the test statistic (e.g. *F*, *t*, *r*) with confidence intervals, effect sizes, degrees of freedom and *P* value noted<br>*Give P values as exact values whenever suitable.*
[x]	[ ]	For Bayesian analysis, information on the choice of priors and Markov chain Monte Carlo settings
[x]	[ ]	For hierarchical and complex designs, identification of the appropriate level for tests and full reporting of outcomes
[x]	[ ]	Estimates of effect sizes (e.g. Cohen's *d*, Pearson's *r*), indicating how they were calculated

Our web collection on statistics for biologists contains articles on many of the points above.

Software and code

Policy information about availability of computer code

Data collection	EPU-v2.2.0
Data analysis	Relion 3.1, Rosetta 3.1, CryoSPARC 2.15, Pymol 2.3.2, Coot 0.89, Phenix 1.18.2, MotionCor2, CTFFIND4.1, UCSF Chimera 1.15, crYOLO 1.7.4, MolProbity 4.1, Amber 20, UCSF ChimeraX 1.3

For manuscripts utilizing custom algorithms or software that are central to the research but not yet described in published literature, software must be made available to editors and reviewers. We strongly encourage code deposition in a community repository (e.g. GitHub). See the Nature Research guidelines for submitting code & software for further information.

Data

Policy information about availability of data

All manuscripts must include a data availability statement. This statement should provide the following information, where applicable:

- Accession codes, unique identifiers, or web links for publicly available datasets
- A list of figures that have associated raw data
- A description of any restrictions on data availability

All data produced or analyzed in this study are included in the main text or the supplementary materials. A reporting summary for this article is available as a Supplementary Information file. The cryo-EM density maps and atomic coordinates have been deposited in the Electron Microscopy Data Bank (EMDB) and Protein Data Bank (PDB) under accession numbers EMD-32881 and 7WXU for the GPR110/Gq complex; EMD-32882 and 7WXW for the GPR110/Gs complex; EMD-32972 and 7X2V for the GPR110/Gi complex; EMD-32905 and 7WZ7 for the GPR110/G12 complex, EMD-32883 and 7WY0 for the GPR110/G13 complex, respectively.

Field-specific reporting

Please select the one below that is the best fit for your research. If you are not sure, read the appropriate sections before making your selection.

☒ Life sciences ☐ Behavioural & social sciences ☐ Ecological, evolutionary & environmental sciences

For a reference copy of the document with all sections, see nature.com/documents/nr-reporting-summary-flat.pdf

Life sciences study design

All studies must disclose on these points even when the disclosure is negative.

Sample size	For all reporter functional assays, BRET assay and neurite outgrowth assay, three independent experiments (n=3) are carried out and sufficient. ee figure legends for detail.
Data exclusions	No data were excluded
Replication	Key functional experiments (reporter assay, BRET assay and neurite outgrowrh assay) were repeated at least two times independently,all attempts at replication were successful.
Randomization	No Randomization was attempted or needed. Randomization was not necessary as the independent variables to be tested were sufficient for functional interpretation within this study.
Blinding	Blinding was not necessary for structural determination as in this case, cryo-EM captured conformations representing a large amount of individual particles, or most time the major classes of all particles. Also, blinding is not necessary for functional analysis of this study. All experimental data acquired in this study are subjected to statistical analysis when necessary.

Reporting for specific materials, systems and methods

We require information from authors about some types of materials, experimental systems and methods used in many studies. Here, indicate whether each material, system or method listed is relevant to your study. If you are not sure if a list item applies to your research, read the appropriate section before selecting a response.

Materials & experimental systems

n/a	Involved in the study	
☒	☐	Antibodies
☐	☒	Eukaryotic cell lines
☒	☐	Palaeontology and archaeology
☐	☒	Animals and other organisms
☒	☐	Human research participants
☒	☐	Clinical data
☒	☐	Dual use research of concern

Methods

n/a	Involved in the study	
☒	☐	ChIP-seq
☒	☐	Flow cytometry
☒	☐	MRI-based neuroimaging

Eukaryotic cell lines

Policy information about cell lines

Cell line source(s)	sf9 cell line, Invitorgen cat#11496-015; AD-293 cell line, Agilent # 240085
Authentication	No authentication required
Mycoplasma contamination	All cell lines tested are negative for mycoplasma contamination
Commonly misidentified lines (See ICLAC register)	No commonly misidentified cell lines were used

Animals and other organisms

Policy information about studies involving animals; ARRIVE guidelines recommended for reporting animal research

Laboratory animals	Female C57BL/6, Charles River Laboratories (Beijing, China). Mice were housed on a 12-h light/12-h dark cycle, temperature (24 ± 2 ° C) and humidity (50% ± 10%) conditions. Total 6 new born (one day old) mice were used.
Wild animals	Not applicable in this study

Field-collected samples	not applicable in this study
Ethics oversight	Animal experiments were carried out in strict accordance with the Guide for the Care and Use of Laboratory Animals (8th edition) and approved by the Institutional Animal Care and Use Committee of Harbin Institute of Technology (HIT/IACUC).

Note that full information on the approval of the study protocol must also be provided in the manuscript.
